# Physical activity in professional training of physiotherapists

**DOI:** 10.1080/07853890.2024.2446687

**Published:** 2025-01-02

**Authors:** Karel Frömel, Jan Dygrýn, Michal Vorlíček, Lukáš Jakubec, Dorota Groffik, David Smékal, Josef Mitáš

**Affiliations:** a Faculty of Physical Culture, Palacký University Olomouc, Olomouc, Czech Republic; b The Jerzy Kukuczka Academy of Physical Education in Katowice, Katowice, Poland

**Keywords:** Physical activity, professional education, lifestyle, monitoring, wearables

## Abstract

**Background:**

The current negative trend in the physical behavior and lifestyle of the population therefore requires adequate changes in the professional training of physiotherapists.

**Objectives:**

This study aimed to determine the structure and differences in the weekly physical activity (PA) of Czech physiotherapy students, the use of wearables in physiotherapy professional training, and the attitude of physiotherapy students toward PA and the use of wearables in physiotherapy practice.

**Methods:**

Between 2013 and 2022, 412 physiotherapy students participated in a PA-monitoring study using questonnaires International Physical Activity Questionnaire-long form, Motives for Physical Activity Measure-Revise, pedometers, Garmin Vívofit and Axivity AX3 accelerometers.

**Results:**

A retrospective analysis of physiotherapy students PA drew attention to insufficient weekly PA and insufficient achievement of the PA recommendation of at least 60 min five times a week (55% of men and 41% of women). Instrumental PA monitoring allowed analyzing individual daily PA and structure of weekly PA. Highest PA indicated men (14,102 steps/day) and women (12,724 steps/day) of the 1st study year on Tuesday. The lowest PA (9,488 steps/day for men and 8,815 steps/day for women), were observed in the 4th study year on Sundays. The recommended target of 11,000 steps per day was achieved by 40% of the men and 46% of the women. Wearables enhanced participants PA motivation (51%).

**Conclusions:**

The inclusion of weekly PA monitoring in the professional training of physiotherapists ensured a deeper insight into the possibilities of PA monitoring in physiotherapy practice . Students are prepared to use wearables more widely to improve physical therapy practice.

## KEY MESSAGES

- The study demonstrated the need for greater support for physical activity (PA) of physiotherapy students during their studies.
- Incorporating comprehensive weekly PA monitoring into physiotherapy education is essential, especially with the rapid development of wearable technology.
- Monitoring PA using different methods enabled students to better assess the possibilities of using wearables in physiotherapy practice.

## Introduction

### Physical activity in physiotherapy theory and practice

Physical activity (PA) is an integral part of a healthy ­lifestyle and aging [1]. Optimal PA, which limits sedentary behavior (SB) and promotes sleep quality, brings significant health benefits to the youth. These benefits are related to adiposity, cardiometabolic risk factors, cardio-respiratory fitness, muscular physical fitness, well-being, health-related quality of life, mental health, academic performance, and cognitive function [2]. Positive associations between physical exercise and mental health have been found in adolescents [3] and older adults [4]. Despite this evidence of the importance of PA for a healthy lifestyle for all age groups, a global prevalence of insufficient PA [5,6], a decrease in PA with age [7], and an increase in SB with age was registered [8].

Supporting PA in the general population should be a priority of state health policies [9]. Physiotherapists may play a significant role in promoting PA and positive changes in movement behavior and improving the health literacy of all age groups [10,11]. In numerous areas of physiotherapy practice, the effects of physiotherapy programs, combined with effective psycho-educational influences on patients, provide exceptional possibilities to change movement behaviors and support a healthy lifestyle [12], but also in mental health support [13].

Unfortunately, we continue to encounter under-appreciated support for movement behavior in ­physical therapy practice, even though there are numerous calls for PA promotions in physiotherapy [14]. Physiotherapists under-utilize the possibility of PA in therapy and its intensive use remains a significant challenge [15].

### The use of wearables in physiotherapy

One of the current options for supporting PA in physiotherapy practice is the more effective use of wearables to monitor movement behaviors [16]. There is tremendous potential for interactive wearable technologies in rehabilitation, especially with the help of tele-rehabilitation models [17], when setting goals and planning treatment using home physical exercises [18]. The results of these interventions led to the cautious conclusion that wearables can have a positive impact on the number of steps per day taken by patients after total knee or hip arthroplasty [19]. Moreover, biofeedback rehabilitation has positive effects on dynamic balance and neurological diseases associated with mobility disorders [20]. Wearables provide better possibilities for disease assessment and treatment of patients with Parkinson’s disease symptoms [21]. From a broader perspective, wearables can be effective in the treatment and rehabilitation of numerous chronic diseases [22].

Despite this substantial evidence of the contribution of wearables in physiotherapy, their long-term positive effects on the subsequent post-therapeutic life of patients remain insufficiently proven. Numerous studies have shown that big data extracted from wearables may transform our understanding of population health dynamics and predict trends in the effective use of wearables [23]. Among the challenges to the use of wearables in clinical practice, Lang et al. [24] indicated busy clinical environments, lack of quality information, measurement inaccuracies, differences in research needs, user-friendliness for physiotherapists and patients, and insufficient choice of wearables for specific patient needs. Blumenthal et al. [25] stated that physiotherapists have a positive attitude toward the potential for using mobile or wearable technology; however, greater support for wearables will require proof of patient satisfaction, adherence, and tangible clinical outcomes.

### Physical activity and its motivation in physiotherapy students

Support for the effective use of PA in physiotherapy should primarily focus on supporting PA in the professional training of physiotherapists [26]. Badau et al. [27] found very low PA among Romanian physiotherapy ­students, and the main motivation dimensions were ­enjoyment, competence/challenge, and fitness/health. Physiotherapy students cite physical exertion as the main obstacle to performing PA as only 37.5% of the students engage in vigorous PA [28]. The authors are highly critical of PA in physiotherapy students, calling for the monitoring of the PA levels of physiotherapists across South Africa and the inclusion of PA in the curriculum.

Several physiotherapists lack sufficient PA knowledge. In a study by Barton et al. [29], 60% of physiotherapists’ PA guidelines were for adults, 53% for children, and 37% for older adults. Stead et al. [30] found that UK physiotherapists had limited PA guideline awareness. Similarly, Australian physiotherapists have poor knowledge of the Australian PA and SB guidelines and infrequently promote PA [31]. Ryu et al. [32] critically evaluated the knowledge level of physical therapy students regarding PA recommendations.

The professional training curriculum for physiotherapy students should expand PA education to include competencies to promote PA [15]. Greater support for PA in students should be provided by integrating behavioral medicine competencies into physiotherapy [33]. In this context, it is important to ‘ground’ the physiotherapy curriculum in a wider context of a healthy lifestyle. Related to this is the assumption that physically active physiotherapists have more self-confidence and actively support PA [34].

The professional training of physiotherapists should respond to the growing role of new technologies, which, among other things, means deepening research, education, and the practical use of wearables. Therefore, in our study, we present an option for improving the position of movement behavior in professional physiotherapy training.

This study aimed to determine the structure and differences in the weekly PA of Czech physiotherapy students, the use of wearables in physiotherapy professional training, and the attitude of physiotherapy students toward PA and the use of wearables in physiotherapy practice.

***Research questions***

- What is the PA level of physiotherapy students?
- What are the differences in the PA of men and women and the PA of the 1st and 4th year students?
- How do physiotherapy students evaluate the monitoring of weekly movement behavior using wearables?

## Methods

### Study design

This was a cross-sectional study over 10 years and is based on the subjective self-awareness theory [35]. This theory emphasizes the importance of self-awareness, awareness of the association between ­feelings of satisfaction and movement behavior, and awareness of the PA benefits in physiotherapy practice. Understanding the positive and negative effects of mobile or wearable devices is crucial for more effective use of technology in physiotherapy practice [25]. Therefore, we consider it important to respect the progress in the development of the technology acceptance model, emphasizing perceived usefulness and perceived ease of use of technology [36].

### Participants and procedures

A total of 412 physiotherapy students (110 males and 302 females) at the Faculty of Physical Culture of the Palacký University in Olomouc, Czech Republic, participated in the research during October and November from 2013 and 2022. Every year, 1st and 4th year students, who were able to complete weekly PA monitoring, participated in the study. On average, 5–10% of the students could not participate for health reasons each year. Furthermore, 53 students were excluded because they did not meet the requirements set by the measurement methods and the Guidelines for Data Processing and Analysis of the International Physical Activity Questionnaire (IPAQ) short and long forms (IPAQ Research Committee, 2005).

**Figure 1.**
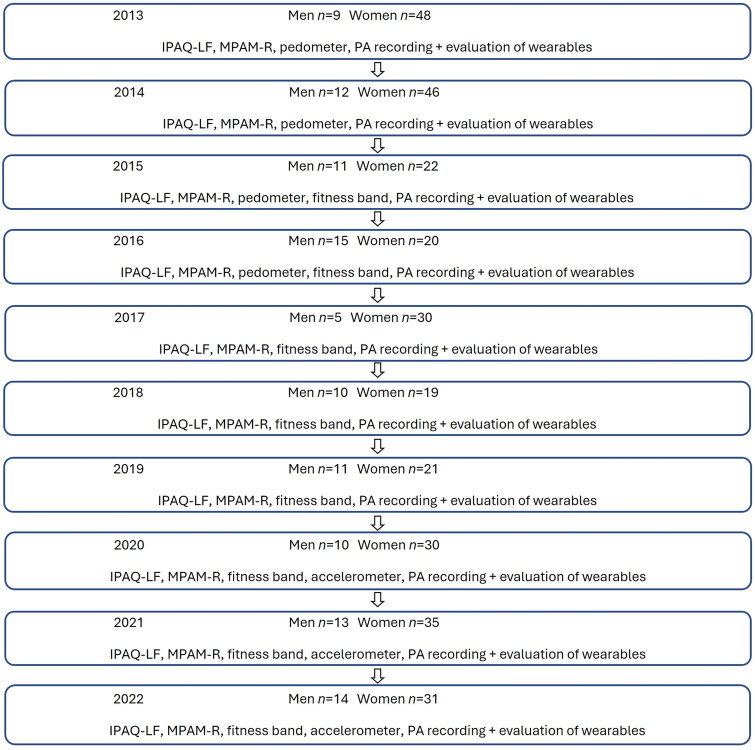
Study design – years and measurements.

Due to the different number of students and the smaller number of boys, we did not analyze gender differences in each research year. Participants’ involvement in each research year, with an overview of the methods, is presented in Figure 1.

### Measurements

In every research year, participants registered on the web application ‘International Database for Research and Educational Support’ (INDARES) (www.indares.com), and all the data were recorded there. This allowed the participants to analyze individual results. The same three-member research team provided information on the progress of the research throughout its duration. In the introductory session, the students as a group received a brief introduction to the research process, questionnaire completion, and the PA observation method. Students were trained on handling wearables and connecting to the Garmin Connect app on a smartphone. In the weekly PA record, they reported wake up time and the time before falling asleep, number of steps and minutes of moderate to vigorous PA (MVPA). They were also able to comment on their motivation for PA, recommendations for PA, advantages and disadvantages of wearables and their use in physiotherapy practice. Students’ views on PA and the potential use of wearables in physiotherapy practice were subjected to axial and selective coding to define the main categories of assessment [37].

The wearables were worn by the students continuously throughout the week on the wrist of the non-dominant hand. In written materials, students received a detailed timeline for completing the questionnaires and PA monitoring.

We determined the level and structure of weekly PA using the standardized Czech version of the IPAQ [38,39]. The IPAQ includes PA types (school-related, transportation, housework, house maintenance and caring for family, recreation, sports, and leisure time), PA intensity (vigorous, moderate, and walking), and sedentary time. Compared with the IPAQ scoring protocol, the MET-min of vigorous PA was multiplied by six, instead of eight, and the maximum MET-min/week was limited to 16,000 MET-min/week. The recommendation of at least five times a week of not less than 60 min of moderate to vigorous PA per day, at least three times a week and not less than 20 min of vigorous PA (VPA) per day, and at least three times a week was in agreement with the global PA recommendations [40].

To determine PA motivation, we used the Czech version of the questionnaire ‘Motives for Physical Activity Measure-Revise’ (MPAM-R) [41,42]. The questionnaire consisted of 30 items in five categories – enjoyment, competence, appearance, fitness, and social factors. Categories were assessed on a 7-point Likert scale (1 = not at all true for me to 7 = very true for me).

To monitor weekly PA, we used a Digi-Walker SW-700 pedometer (Yamax Co., Yasama Corp., Tokyo, Japan) from 2013 to 2017 and Garmin Vívofit 1 or 3 fitness bands from 2018 to 2022. The differences between the Yamax pedometer and the Garmin Vívofit 1 and 3 were not significant in the assessment of average steps/day [43,44]. From 2018 to 2022, Axivity AX3 accelerometers were used to measure PA volume and intensity, sedentary time, and sleep quality. All the participants received individual feedback regarding the questionnaires and wearables results. In addition, the mean group results and the comparisons of individual results were analyzed sensitively to maintain anonymity. Based on the study of physiotherapists at the Faculty of Physical Culture and the awareness of the importance of PA in physiotherapy practice, we set a more demanding recommendation for PA in a study of 11,000 steps/day, according to the recommendation for adolescents [45].

### Statistical analysis

We used the statistical program Statistica version 14.0.0.15 (StatSoft, Prague, Czech Republic) and R Software for data processing and statistical analyses. We used basic descriptive statistics to characterize the set and assess the normality with Kolmogorov-Smirnov and Lilliefors tests. The Kruskal-Wallis ANOVA test was used to evaluate the types of weekly PA. We analyzed the differences in the mean steps/day between days in a week and between the 1st and 4th year of study using repeated-measures ANOVA with Scheffe’s post-hoc test. Box’s M and Mauchly’s sphericity tests were used to determine whether the ANOVA assumptions were violated. We assessed the achievement of PA recommendations according to cross-tabulation. The *ŋ*^2^ effect size coefficients were evaluated as follows: 0.01 ≤ *ŋ*^2^ < 0.06 small effect size, 0.06 ≤ *ŋ*^2^ < 0.14 medium effect size, *ŋ*^2^ ≥ 0.14 large effect size. The level of statistical significance was set at *p* < 0.05 and a logistic significance of 10 p.p.

## Results

### Sample characteristics

The basic characteristics of students in the 1st and 4th years are shown in Table 1. The involvement of students in organized PA is important for the analysis of students’ PA. There was significantly lower participation in organized PA of 4th-year students compared to 1st-year students, especially among women (*χ*^2^ = 7.56; *p* = 0.006; *η*^2^ = 0.069), however, men demonstrated a 10.1 p.p. decrease, which is logically significant.

**Table 1.** Sample characteristics.

Gender	Years of study	*n*	Age (years)		Weight (kg)		Height (cm)		BMI (kg·m^−2^)		Organized PA
			*M*	*SD*	*M*	*SD*	*M*	*SD*	*M*	*SD*	(%)
Men	1st	61	20.5	1.4	78.5	9.5	182.6	6.2	23.5	2.4	67.2
	4th	49	21.8	2.2	77.8	7.7	182.7	5.7	23.3	2.0	57.1
Women	1st	122	20.2	1.0	61.7	6.3	169.4	5.9	21.5	1.9	69.7
	4th	180	21.9	2.2	61.0	7.5	168.6	6.3	21.4	2.3	53.9

BMI = Body Mass Index; *M =* mean; *SD* = standard deviation; PA = physical activity.

### Types of weekly PA of men and women in the 1st and 4th year of study according to the IPAQ questionnaire

**Figure 2.**
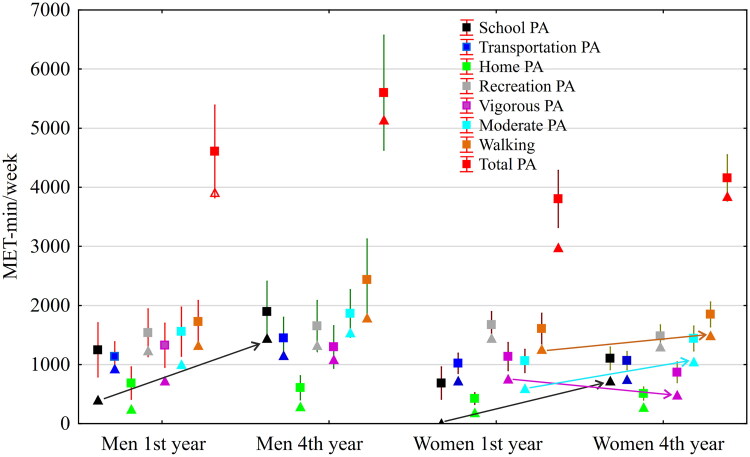
Differences in types of PA in men and women in the 1st and 4th year of study (Mean and Median) according to the IPAQ questionnaire.

Differences between men in 1st year and in 4th year and equally between women in 1st year and in 4th year of study were significant in school PA (*H*_(3,412)_ = 35.95, *p* < 0.001, *η*^2^ = 0.081). And this is significantly in favor of men 4th year (*p* = 0.009) and women 4th year (*p* < 0.001) (Figure 2). Furthermore, moderate PA (*H*_(3,412)_ = 16.76, *p* < 0.001, *ŋ*^2^ = 0.034) favored women in the 4th year (*p* = 0.008). Similarly, walking (*H_(3,412_*_)_ = 7.53, *p* = 0.057, *η*^2^ = 0.011) favored women in the 4th year (*p* = 0.034) as compared with women in the 1st year. However, vigorous PA (*H*_(3,412)_ = 12.34, *p* = 0.006, *ŋ*^2^ = 0.023) favored women in the 1st year as compared with women in the 4th year (*p* = 0.028). In total PA, men in the 4th year achieved *Mdn ± IQR* = 5,136 ± 4,647 MET-min/week, while women in the 4th year achieved *Mdn ± IQR* = 3,839 ± 3,219 MET-min/week (*p* = 0.006).

Regarding the recommendation for 3 × 20 min of VPA (Table 2), there are significant differences between the 1st (46.7%) and 4th year (31.7%) women (*χ*^2^ = 7.01; *p* = 0.008; *ŋ*^2^ = 0.023). Significantly more men in the 4th year met the recommendations for 3 × 20 min of VPA than women in the 4th year (*χ*^2^ = 6.28, *p* = 0.012, *ŋ*^2^ = 0.027). Regarding the recommendation for 5 × 60 min of MVPA, significant differences were found between men and women in the 1st year (*χ*^2^ = 4.50, *p* = 0.034; *ŋ*^2^ = 0.025).

**Table 2.** Achievement of the recommendations for vigorous and vigorous to moderate physical activity in men and women in the 1st and 4th year of study.

Recommendation	Men (%)		Women (%)		*χ* ^2^	*p*	*η* ^2^
	1st year of study	4th year of study	1st year of study	4th year of study			
3 × 20 minute VPA	45.9	51.0	46.7	31.7	10.77	0.013	0.026
5 × 60 minute MVPA	52.5	59.2	36.1	43.9	9.37	0.025	0.023

PA = physical activity; VPA = vigorous physical activity; MVPA = moderate to vigorous physical activity; *χ*^2^ = Pearson’s chí-squared test; *p* = level of significance; *η*^2^ = effect size coefficient.

In summary, 48.2% of the men and 37.8% of the women met the recommendation for 3 × 20 min of VPA (*χ*^2^ = 3.64, *p* = 0.056, *ŋ*^2^ = 0.009), while 55.5% of the men and 40.7% of the women met the recommendation of 5 × 60 min of MVPA (*χ*^2^ = 7.07, *p* = 0.008, *ŋ*^2^ = 0.017).

### Structure of weekly PA of men and women in the 1st and 4th year according to wearables

There were significant differences between individual days of the week (*F*_(6,2448)_ = 14.63, *p* < 0.001, $\eta_{p}^{2}\;$= 0.035). Men (14,102 steps/day) and women (12,724 steps/day) from the 1st year had significantly higher PA on weekends than on other days of the week (*p* < 0.001). Students had the lowest PA on Sunday, compared to Monday (*p* = 0.016), weekends (*p* < 0.001), wedges (*p* = 0.032), and Friday (*p* = 0.021). There were significant differences between the days of the week between 1st and 4th-year students (*F*_(6,2448)_ = 6.26, *p* < 0.001, $\eta_{p}^{2}$ =0.015).

**Figure 3.**
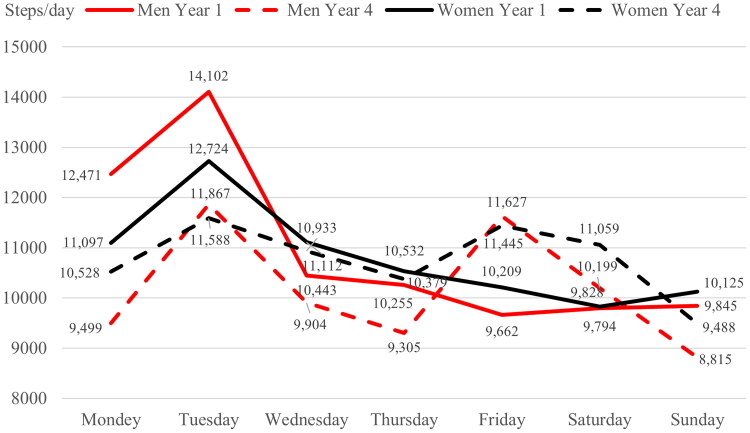
Average steps/day of men and women in the 1st and 4th year of study.

According to gender, there were significant differences between the days of the week (Days x Gender) *F*_(18,2448)_ = 2.92, *p* < 0.001, $\eta_{p}^{2}$ =0.021. However, gender differences on individual days of the week were not significant (Figure 3).

A total of 40.0% of the men and 45.7% of the women met the recommendation of 11,000 steps/day (*χ*^2^ = 1.06; *p* = 0.303; *η*^2^ = 0.003) and gender differences were not significant. The recommendation of 11,000 steps/day was significantly more likely to be met by 57.4% of the men in the 1st year versus 22.5% of the men in the 4th year on Monday (*p* < 0.001) (Table 3), by 62.3% of the women in the 1st year and 46.7% of the women in the 4th year on Monday (*p* = 0.007), and by 42.2% of the women in the 4th year versus 22.5% of the men in the 4th year on Monday (*p* = 0.011).

**Table 3.** Meeting the recommendation of 11,000 steps/day for men and women in the 1st and 4th year of study.

Recommendation 11,000 steps/day	Men %		Women %		*χ* ^2^	*p*	*η* ^2^
	1st year of study	4th year of study	1st year of study	4th year of study			
Monday	57.4	22.5	47.5	42.2	14.52	0.002	0.035
Tuesday	63.9	46.9	62.3	46.7	10.68	0.013	0.026
Wednesday	44.3	30.6	46.7	40.0	4.12	0.249	0.010
Thursday	36.1	26.5	42.6	41.1	4.43	0.219	0.011
Friday	32.8	51.0	41.8	51.1	7.53	0.057	0.018
Saturday	37.7	38.8	36.9	46.7	3.56	0.313	0.009
Sunday	34.4	22.5	36.1	33.3	3.05	0.384	0.007

*χ*^2^ = Pearson’s Chi-squared test; *p* = level of significance; *η*^2^ = effect size coefficient.

Table 4 presents comparative data on movement behaviors measured by wrist-worn Axivity AX3 accelerometers between the 1st and 4th-year students. The results show that 4th-year students had a higher average SB (*p* = 0.011) and lower sleep time (*p* = 0.020) compared to 1st-year students.

**Table 4.** Differences in movement behaviors measured by Axivity AX3 accelerometers between students of the 1st and 4th year of study.

	Year of study		
Movement behaviors	1st (*n* = 32)	4th (*n* = 31)	*p*
SB	676.4 (63.7)	724.3 (81.1)	0.011
LPA	168.3 (42.6)	151.6 (32.0)	0.085
MVPA	136.0 (31.9)	128.1 (62.8)	0.531
Sleep	467.2 (44.6)	440.0 (46.1)	0.020
Sleep efficiency	90 (5)	88 (6)	0.190

Values are presented as mean (SD). The differences between students of 1st and 4th year were evaluated using the Student’s *t*-test. Significance level: *p* < 0.05. SB = sedentary behavior; LPA = light physical activity; MVPA = moderate-to-vigorous physical activity.

**Figure 4.**
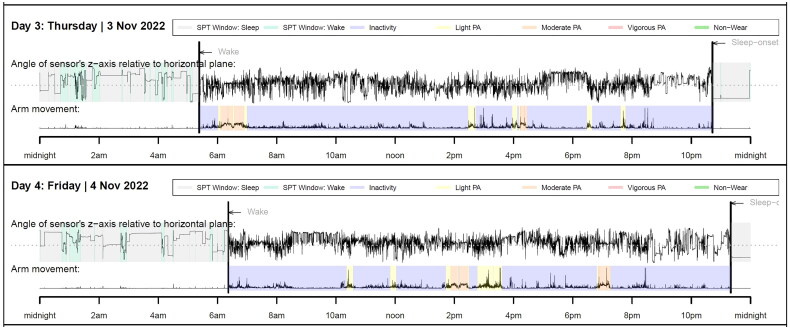
Example of individual feedback of movement behavior from Axivity AX3 provided to each student.

Figure 4 illustrates an example of personalized feedback on daily movement behaviors provided to physiotherapy students. Feedback visualization is structured to reflect a typical day, from wake-up time to sleep onset. It uses a color-coded system to denote different types of activities – SB (Inactivity), light PA, moderate PA, and VPA. It serves as an educational tool aiding physiotherapy students in self-assessing daily movement behaviors and understanding the importance of balancing different PA intensities with adequate rest for optimal health. This is a practical example of the translation of the accelerometer data into actionable personal insights.

### PA motivation by gender and year of study

**Figure 5.**
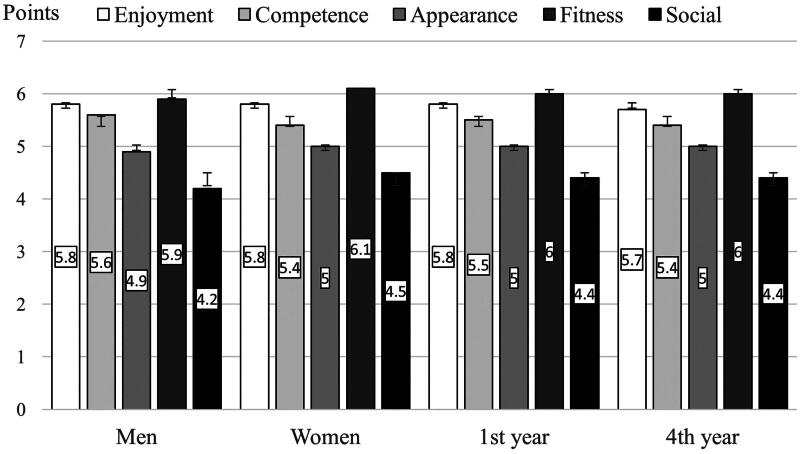
Differences in the dimensions of motivation for PA according to gender and year of study.

There were no significant differences between men, women, 1st, and 4th year of study in the dimension of motivation enjoyment/interest (*H*_(3,412)_ = 4.15, *p* = 0.245, *ŋ*^2^ = 0.003), competence (*H*_(3,412)_ = 3.29, *p* = 0.349, *ŋ*^2^ = 0.001), appearance (*H_(3,412_*_)_ = 1.16, *p* = 0.762, *ŋ*^2^ = 0.001), fitness (*H*_(3,412)_ = 4.02, *p* = 0.259, *ŋ*^2^ = 0.002), or social (*H*_(3,412)_ = 4.04, *p* = 0.257, *ŋ*^2^ = 0.003) (Figure 5). Men and women were most motivated by the fitness dimension and least motivated by the social dimension. Social motivation was significantly lower for both men and women than other motivation types (*p* < 0.001). More motivated students showed higher total PA (4,813 MET-min/week) than less motivated students (3,777 MET-min/week) (*p* < 0.001).

### Evaluation of physiotherapy students’ attitudes toward PA monitoring and the use of wearables in physiotherapy practice

In summary, 51.3% of the 1st year and 52.1% of the 4th year students expressed that wearables motivated them to engage in higher PA (*p* = 0.908). Furthermore, 94.6% of the 1st year and 95.0% of the 4th year students said that providing PA recommendations could support efforts to increase PA (*p* = 0.859). It is noteworthy that 23.1% of the students considered mobile phones to be more suitable than wearables for immediate PA information.

*When evaluating Garmin fitness bands at a general level, students most often reported:*

*Benefits:*

- The motivation to increase PA for a healthy lifestyle and reduce weight (65.8%).
- Immediate feedback on PA and SB (35.0%).
- The possibilities of setting PA goals and checking achievements (34.2%).
- Ease of use (20.8%; e.g. ‘Based on this PA monitoring experience, I will buy a wearable’).

*Disadvantages:*

- Inaccuracies in PA measurements (21.7%).
- Discomfort, especially after 24 hours (12.5%).
- Negative skin reactions (8.3%).
- Technical issues with synchronization or batteries (7.5%).

*Based on the experience of physiotherapy practice with patients and clients, 4th-year students reported:*

- Better motivation and PA options for certain patients (e.g. ‘I know from my own experience that patients who use smartwatches are more active and motivated for PA’).
- Good experience with group motivation for PA, especially with ‘competitive’ patients.
- Possibilities for better assessment of additional homework, especially walking activities.
- Improving the quality of physiotherapy for patients with increased body weight (e.g. ‘Wearables are a great assistance to me and prove useful in my work with patients who are trying to reduce weight’).
- Good experience of using wearables in older adults if technological issues are eliminated.
- Wearables enable a better individual approach to patients (e.g. ‘Wearables allow me to improve the quality of therapeutic interventions and better respect patient’s individuality’).
- Some students see the possibilities of wearables as special devices, enabling the use of distance therapy/tele-rehabilitation.
- Wearables make it possible to deepen PA knowledge and data concerning eating habits, energy intake, and expenditure.
- Wearables enable better awareness of feelings of well-being and life satisfaction and limit distress.

*Students made certain critical comments:*

- Wearables are purchased only by PA-motivated patients.
- Too much orientation to information from the wearables is sometimes at the expense of the perception of ‘self’ and one’s body.

*Most comments were about wearing wearables during physiotherapy:*

- This poses a problem for patients who are not used to wearing watches.
- Critical comments were about an unsatisfactory display, especially in elderly patients.
- It is an additional ‘concern’ during physiotherapy work (e.g. ‘It bothers me at work’, ‘Most of us are used to taking everything off of our hands when at work’).
- It bothers me when using some methods (e.g. ‘It bothers me and limits me when performing myofascial techniques’).

The use of the axis accelerometer was evaluated positively for sleep quality, albeit with minor inaccuracies, and a better overview of PA intensity. However, critical remarks were made regarding the lack of feedback on movement and SB.

## Discussion

### PA in physiotherapy students

The PA level of physiotherapy students, as determined by the IPAQ questionnaire and PA monitoring using wearables, indicated insufficient PA within a 10-year follow-up. These findings correspond with previous findings [27,28,46,47]. We did not find a study that resulted in a significantly positive PA assessment of physiotherapy students. Earlier research states that almost half of physiotherapy students were not sufficiently physically active [48]. According to Kgokong and Parker [28], only 37.5% of the physiotherapy students showed VPA. Similar results in VPA in 1st year of physiotherapy study were reported by Boguszewski et al. [46] for 36% of the women and 52% of the men. However, more than half of those surveyed did not engage in any PA outside the curriculum. Critical evaluation of the PA level of physiotherapy students in our group needs to be considered concerning the insufficient PA levels of youth worldwide [7] and the methodologically weak evidence and inconsistencies of PA assessment and monitoring methods [49].

It must be studied to what extent the insufficient PA of physiotherapy students influenced by the insufficient PA and barriers to practicing PA by university students in general [50,51] or the insufficient PA of physiotherapists in practice. Lowe et al. [14] reported that only 38% of physiotherapists met the recommendation of 5 × 30 minutes of moderate PA. More favorable results were found by Neil-Sztramko et al. [52], who found that 99% of the physiotherapists in British Columbia met the PA recommendation; however, when using accelerometers, the number dropped to 58%, still an exceptionally high level of compliance. The authors drew attention to the poor agreement between PA assessment using questionnaires and accelerometers, however, they considered physiotherapists to be a physically active group. These differences between the IPAQ and axial accelerometer compliance are consistent with the results of this study. There remains a lack of evidence that physiotherapists with higher PA levels promote better PA in their patients and are more successful in health prevention. Notably, Kunstler et al. [53] stated that it is important (47%) or extremely important (29%) for patients that a physiotherapist provides advice to help them increase their PA levels.

Our results indicated a relatively high average steps/day for men and women in the 1st and 4th year of study (11,588–14,102 steps/day). We found the most significant involvement in PA by physiotherapy students on Tuesday in a previously published study in the same educational environment [48]. We found the weekly educational program and the distribution of study duties to be the main cause of this finding.

### Technology in physiotherapy practice

Only 52% of the students reported that wearables motivated them to perform more PA. This was mainly influenced by the long-term use of wearables and mobile phones, and the fact that PA monitoring was carried out using two different devices. However, the positive attitude of most students toward wearables was obvious and they were hopeful for its greater use in future practice.

The specificity of the demanding professional physiotherapy education of the students participating in the study was reflected in the strict evaluation of the lack of accuracy of the wearables, which is essential for physiotherapy practice. Numerous studies have reported claims to increase the objectivity and accuracy of wearables in clinical practice [24,54]. Treacy et al. [55] found that the StepWatch and Fitbit One wearables, worn on the ankle, maintained accuracy in individuals who walked slowly with shorter strides, while other devices were less accurate in these individuals. Objective measurement devices are important for monitoring patients during rehabilitation and home exercises [56]. The views of certain research participants regarding the difficulty of using technology in physiotherapy practice correspond to the findings of McGrath et al. [57] who stated that the use of technology was outside or on the boundary of their scope of practice.

### PA motivation for physiotherapy students

In the MPAM-R questionnaire, students expressed that they were most motivated to engage in PA in the fitness/health dimension and least motivated in the social dimension. Similar results were found by Badau et al. [27], whereby enjoyment, fitness/health, and competence/challenges were the main PA motivations for Romanian physiotherapy students, while social had the lowest motivational effect. The PA motivation of physiotherapy students is important because more motivated students show more weekly PA, which was confirmed by Mahony et al. [58]. Understanding and respecting the associations between motivation types and PA can support PA enjoyment, increase PA levels, and aid PA recommendations [59]. This could support the success of physiotherapy students in the more effective application of PA in their practice. Moore et al. [60] drew attention to the fact that in elderly patients, motivation for wearable use was both intrinsic and extrinsic, encompassing several aspects of user experience, which appeared to be more important than actual device features.

### Incentives to support PA in the professional training of physiotherapists

- Ensure that all physiotherapy students undergo at least weekly monitoring of their movement behaviors, especially PA.
- Provide the option of choosing wearables in practice, respecting the specificities of different types of therapy and patients’ age.
- To expand the possibility of physiotherapy students using physical assessment within the curriculum for the entire study period.
- To familiarize physiotherapy students with the possibilities of using wearables in tele-rehabilitation to improve the quality of control and cooperation with patients when performing home physical exercises.
- To deepen the knowledge of physiotherapy students about the associations between PA activity types, PA motivation types, and diagnosis methods.
- Familiarize students with the possibility of using wearables in group therapy.

### Strengths and limitations

The uniqueness of this 10-year cross-sectional study lies in the stable and natural educational setting of the professional training of physiotherapists and the same composition as the research team. The connection between education, research, and physiotherapy is positive.

A limitation of this study is that the research was conducted in the context of a curriculum, so consent to the research may have been influenced by students’ efforts to responsibly complete course requirements. However, once PA monitoring was initiated, students were allowed to withdraw from the research at any time and complete other educational tasks. However, a positive limitation is that the research was conducted under strict conditions, even with pandemic restrictions. Another study limitation is the necessity of changing wearables, especially Garmin wristbands for pedometers, for time and educational reasons. In individual years, it was not possible to ensure the same number of participants and gender differences, and in the 4th year, it was not possible to exclude students who had already participated in the research in the 1st year. There were 40–50% of such students in each study year.

### Future studies

Future studies should focus on the use of wearables in various physiotherapy practices. The use of wearables in physiotherapy manifests as a long-term positive change in patients’ lifestyles.

This study highlights the gaps in physiotherapy students’ PA and the positive and negative effects of using wearables in training and physiotherapy practice. Students’ positive attitudes toward wearables for personal use and physiotherapy practices were confirmed. The study further confirmed the necessity of including at least one week of comprehensive monitoring of movement behavior using wearables in both bachelor’s and master’s programs. Interdisciplinary integration and expansion of the PA theory and movement behavior monitoring in the physical therapy curriculum are desirable. Curricular changes should support the expansion and improvement of PA settings for physiotherapy students, namely those PA types that are essential from the viewpoint of physiotherapy.

## Supplementary Material

Figure_Captions.docx

## Data Availability

The data that support the findings of this study are available from the corresponding author Josef Mitáš upon reasonable request.
