# Supplementary material for: Physical activity in professional training of physiotherapists
Source: Ann Med. 2025 Jan 2;57(1):2446687. doi: 10.1080/07853890.2024.2446687 (PMC11703062; doi:10.1080/07853890.2024.2446687)
Supplement: Figure_Captions.docx [file IANN_A_2446687_SM3230.docx]

**Figure Captions:**

**Figure 1.** Study design – years and measurements.

**Figure 2.** Differences in types of PA in men and women in the 1^st^ and 4^th^ year of study (Mean and Median) according to the IPAQ questionnaire.

**Figure 3.** Average steps/day of men and women in the 1^st^ and 4^th^ year of study.

**Figure 4.** Example of individual feedback of movement behavior from Axivity AX3 provided to each student.

**Figure 5.** Differences in the dimensions of motivation for PA according to gender and year of study.
